# Saliva sample for detection of SARS-CoV-2: A possible alternative for mass testing

**DOI:** 10.1371/journal.pone.0275201

**Published:** 2022-09-28

**Authors:** Olumuyiwa Babalola Salu, Iorhen Ephraim Akase, Roosevelt Amaobichukwu Anyanwu, Mercy Remilekun Orenolu, Maryam Abiodun Abdullah, Temie Giwa-Tubosun, Sodiq Abiodun Oloko, Ayomide Michael Oshinjo, Aisha Ajoke Abiola, Kolawole Solomon Oyedeji, Sunday Aremu Omilabu

**Affiliations:** 1 Centre for Human and Zoonotic Virology, Central Research Laboratory, College of Medicine of the University of Lagos, Idi-Araba, Lagos, Nigeria; 2 Department of Medical Microbiology and Parasitology, College of Medicine of the University of Lagos, Idi-Araba, Lagos, Nigeria; 3 Department of Medicine, Infectious Disease Unit, Lagos University Teaching, Idi-Araba, Lagos State, Nigeria; 4 Research and Development Unit, LifeBank, Yaba, Lagos, Nigeria; 5 Department of Medical Laboratory Science, College of Medicine of the University of Lagos, Idi-Araba, Lagos, Nigeria; University of Zambia, ZAMBIA

## Abstract

Molecular diagnostic testing has played a critical role in the global response to the novel Coronavirus disease (COVID-19) pandemic, since its first outbreak in late 2019. At the inception of the COVID-19 pandemic, nasopharyngeal swab sample analysis for COVID-19 diagnosis using the real-time polymerase chain reaction (RT-PCR) technique was the most widely used. However, due to the high cost and difficulty of sample collection, the number of available sample types for COVID-19 diagnosis is rapidly increasing, as is the COVID-19 diagnostic literature. The use of nasal swabs, saliva, and oral fluids as viable sample options for the effective detection of SARS-CoV-2 has been implemented successfully in different settings since 2020. These alternative sample type provides a plethora of advantages including decreasing the high exposure risk to frontline workers, enhancing the chances of home self-sampling, reducing the cost, and significantly increasing testing capacity. This study sought to ascertain the effectiveness of Saliva samples as an alternative for COVID-19 diagnosis in Nigeria. Demographic data, paired samples of Nasopharyngeal Swab and Drooling Saliva were obtained from 309 consenting individuals aged 8–83 years presenting for COVID-19 testing. All samples were simultaneously assayed for the detection of SARS-CoV-2 RdRp, N, and E genes using the GeneFinder^™^ COVID-19 Plus RT-PCR test kit. Out of 309 participants, only 299 with valid RT-PCR results comprising 159 (53.2%) males and 140 (46.8%) females were analyzed in this study using the R Statistical package. Among the 299 samples analyzed, 39 (13.0%) had SARS-CoV-2 detected in at least one specimen type. Both swabs and saliva were positive in 20 (51.3%) participants. Ten participants (25.6%) had swab positive/saliva-negative results and 9 participants (23.1%) had saliva positive/swab-negative results. The percentage of positive and negative agreement of the saliva samples with the nasopharyngeal swab were 67% and 97% respectively with positive and negative predictive values as 69% and 96% respectively. The findings indicate that drooling saliva samples have good and comparable diagnostic accuracy to the nasopharyngeal swabs with moderate sensitivities and high specificities.

## Introduction

A novel coronavirus disease was identified in Wuhan, China, in December 2019 [1]. The infectious agent was later named to be Severe Acute Respiratory Syndrome Coronavirus-2 (SARS-CoV-2). COVID-19 became a global pandemic in March 2020 with a fatality rate of 2–3% [2]. This ongoing COVID-19 pandemic has had an unprecedented impact on the healthcare, economic and social structure across the geographic regions of the world [3, 4]. Globally, there have been over 610 million laboratory-confirmed cases of COVID-19 and about 6.5 million deaths (as of 5^th^ September 2022) and widespread testing remains a major tool in identifying individuals infected with SARS-CoV-2as it influences the management and control strategies in combating the pandemic [4]. To effectively control the COVID-19 outbreak, it is essential to identify a convenient yet effective means of sampling and diagnosis.

Specialized trained personnel or healthcare workers (HCWs) are required to perform oropharyngeal swabs (OPS) and nasopharyngeal swabs (NPS) sample collection with increasingly close contact with the patients. This sample collection method leads to an increased biosafety risk to healthcare professionals via aerosol droplet transmission coupled with reported cases of bleeding and a higher incidence of discomfort for patients [5]. However, since the SARS-CoV-2 RNA has been detected in the saliva of infected patients and oral transmission of COVID-19 has also been suggested [6–9]. Therefore, the saliva sample could be a good alternative as it’s been shown to be an effective biological sample for carrying out viral detection methods [10]. The use of Saliva samples for the detection of SARS-CoV-2 had already been launched in some niches and the US Food and Drug Administration had given an emergency use authorization for saliva-based protocol [11–15]. Saliva sample collection is also a less traumatic/invasive procedure, particularly in pediatric patients [16].

Based on these issues and others including the need for faster mass testing while managing limited resources, it is critical to find a safe, viable, and cost-effective sample collection method. Saliva seems to be a potentially viable substitute with comparable accuracy and reliability to pharyngeal swab collection which can easily be gotten from patients’ passive drool into a sterile container [17, 18]. Aside from these benefits, saliva samples can be collected by the patients themselves without the intervention of any professional. With a self-sample collection structure, personal protective equipment which is increasingly scarce and expensive will only be needed in the direct provision of care. This reduces strain on available human and financial resources and increases capacity for testing [18].

There is a growing wealth of data with varying diagnostic accuracy on the successful use and implementation of saliva samples for SARS-CoV-2 detection using RT-PCR techniques around the world [19–31]. The collection methods and timing of saliva greatly influence the outcome of the testing depending on the collection method. Saliva is a complex bio-mixture that can consist of salivary gland secretion, gingival crevicular fluid, sputum, and/or mucosal transudate, in varying proportions serving as shortcomings to the testing outcomes [32]. Therefore, these shortcomings in the use of saliva samples need to be considered during testing. Also, some sick or dehydrated patients, who produce thick saliva samples. The saliva samples might be difficult to pipet during sample analysis and require that the technician handling such must be well experienced in dealing with such samples [33, 34]. Special care should also be taken when dealing with children’s saliva samples since a lower sensitivity has been reported [34–36]. This study was carried out to assess the diagnostic efficacy and sensitivity of self-collected saliva specimens as compared to Nasopharyngeal swabs for the detection of SARS-CoV-2 in Lagos University Teaching Hospital, Lagos State, Nigeria using reverse transcription-polymerase chain reaction (RT-PCR).

## Materials and methods

### Study location/area

This study was carried out at the COVID-19 sampling collection site of the Lagos University Teaching Hospital (LUTH), which is one of the Nigerian Centre for Disease Control (NCDC) and the Federal Ministry of Health (FMoH) designated facilities for the treatment and management of COVID-19.

The Lagos University Teaching Hospital is a tertiary hospital established in 1961 and is located in Idi-Araba, Surulere, Lagos State; the economic hub of Nigeria [37]. The teaching hospital is affiliated with the College of Medicine of the University of Lagos (CMUL) which was established in 1962. The CMUL trains medical/paramedical students, while LUTH provides them with clinical experience. The Lagos University Teaching Hospital is the largest in Nigeria with 761 beds [37] providing medical services to the over 20 million population of Lagos State and other states in the country.

### Study design and population

This was a cross-sectional study conducted between 1^st^ March and 10^th^ April 2021 involving a total of 309 consenting individuals aged 8 to 83 years (mean age = 36.33yrs) who presented for COVID-19 testing at the LUTH sample collection site in Lagos, Nigeria.

### Ethical consideration

Ethical approval was obtained from the Health Research and Ethics Committee of the College of Medicine of the University of Lagos (HREC, CMUL) with approval number CMUL/HREC/11/20/793. Written consent was obtained from adult participants after due explanations and understanding of the purpose of the study. Consent was obtained from the parents/guardian of minors using a similar approach.

### Specimen collection, transportation, handling, and processing

Demographic data, paired samples of Nasopharyngeal Swab in Universal Viral Transport Media (VTM) (309 vials), and Drooling Saliva in sterile containers (309 vials) were obtained from the consenting individuals in this study. Saliva samples were self-collected under the observation of a healthcare worker (sample collector) who subsequently collected the Nasopharyngeal swab. Each participant was asked to work up saliva by gently rubbing the outside of their cheeks and gently spitting without coughing or clearing their throats into a sterile universal container.

Samples were transported in the cold chain using triple-level packaging, from the collection site in LUTH to the Centre for Human and Zoonotic Virology (CHAZVY), Central Research Laboratory, College of Medicine of the University of Lagos (CMUL), Lagos. This is one of the national reference laboratories designated by the NCDC for COVID-19 testing. Universal safety precautions and handling procedures were carried out as recommended by the Nigerian Centre for Disease Control (NCDC) [38]. All specimen transport containers were disinfected with a 10% hypochlorite solution. Viral agents in specimen aliquots were inactivated in guanidinium-thiocyanate-based lysis buffer at room temperature for 10 minutes before extraction of viral nucleic acid.

### Nucleic acid extraction and real-time reverse transcriptase polymerase chain reaction

The viral nucleic acid from inactivated samples were extracted using a mini spin column RNA extraction kit by Qiagen (Qiagen, Germantown, Maryland, United States) in a Class IIA Biological Safety Cabinet according to the manufacturer´s instructions. The purified ribonucleic acid (RNA) was reverse transcribed into cDNA and amplified, using the GeneFinder COVID- 19 Plus RealAmp RT-PCR test kit in the Biorad CFX96 Real-Time PCR system.

The GeneFinder^™^ COVID-19 Plus RealAmp Kit is a real-time reverse transcription polymerase chain reaction (qRT-PCR) test for the qualitative detection of SARS-CoV-2 RdRp, N, and E genes. The SARS-CoV-2 primer and probe set(s) are designed to detect RNA from the SARS-CoV-2. During the amplification process, the probe anneals to a specific target sequence located between the forward and reverse primers. In the extension phase of the PCR cycle, the 5’ nuclease activity of Taq DNA polymerase degrades the bound probe, causing the reporter dye to separate from the quencher dye, generating a fluorescent signal. Fluorescence intensity is monitored at each PCR cycle by the maestro software of the Biorad CFX96 Real-Time system.

### Statistical analysis

Data generated were entered into excel sheets and analyzed using the R Statistical package. Descriptive statistics for categorical variables were presented as number (percent) and for continuous variables as mean ±standard deviation (SD) or median (interquartile range; IQR). Comparison of means was carried out using Student’s t-test and Wilcon signed rank exact test with statistical significance at 0.05. The percentage of positive and negative agreement, positive predictive value (PPV), negative predictive value (NPV), and a 95% confidence interval (CI) were calculated. Kappa coefficient was used to estimate agreement between nasopharyngeal swabs and saliva RT-PCR test results.

## Results

Although a total of 309 participants were enrolled to provide both swab and saliva specimens, only 299 with valid qRT-PCR results were analyzed in the data set of this study. Samples with valid qRT-PCR are those with target and internal control Cycle threshold values (Ct value) within the kit manufacturer’s acceptable ranges. The 10 samples with invalid qRT-PCR results are both swab and saliva samples which its internal control failed to amplify. The 299 participant specimens analyzed consisted of 159 (53.2%) males and 140 (46.8%) females aged between 8 to 83 years with a mean age of 36.33 (±13.45) years (Table 1). Most of the participants were within active age groups with a frequency of 31.8%, 28.4%, and 18.7% for age groups 20–30, 31–40, and 41–50 years respectively and this was statistically significant (Table 1).

**Table 1:** Age and sex distribution of study participants (n = 299).

Gender		Male	Female	Frequency (%)	X^2^	P value
**Age**	**Age Group**				31.402	2.12E-05
	<20	12	10	22 (7.4)		
	20–30	29	66	95 (31.8)		
	31–40	57	28	85 (28.4)		
	41–50	34	22	56 (18.7)		
	51–60	16	8	24 (8.0)		
	61–70	6	5	11 (3.7)		
	>70	5	1	6(2)		
	**Total**	**159 (53.2)**	**140(46.8)**	**299 (100)**		

The demographic distribution of the two hundred and ninety-nine (299) participants who consented, were enrolled, whose specimens were collected and analyzed by RT-PCR and R Statistical package.

Analysis of the 299 specimens with valid results showed that 39 (13.0%) had detectable SARS-CoV-2 RNA. This comprises 21 (53.8%) males and 16 (46.2%) females with no statistical significance (Table 2). SARS-CoV-2 was detected among all the age groups of the participants with higher frequencies; 22.6%, 20.9%, and 18.2% in age groups <20, 51–60, and 61–70 years respectively with no statistical significance (Table 2).

**Table 2:** Relationship of SARS-CoV-2 positivity with gender and age groups.

Gender	Negative (%)	Positive (%)	Total (%)	X^2^	P value
**Female**	122(46.9)	16(46.2)		**1.46E-30**	**1**
**Male**	138 (53.1)	21 (53.8)			
**Total**	**260 (87)**	**39 (13)**	**299 (100)**		
**Age Groups**					
**<20**	17(77.3)	5 (22.60)	22(100)	**7.3822**	**0.2869**
**20–30**	89(93.6)	6 (6.4)	95(100)		
**31–40**	75(85.9)	12 (14.1)	85 (100)		
**41–50**	48(85.7)	8 (14.3)	56 (100)		
**51–60**	19(79.2)	5(20.9)	24 (100)		
**61–70**	9(81.8)	2(18.2)	11 (100)		
**>70**	5(83.3)	1 (16.7)	6(100)		
**Total**	**260**	**39**			

The overall distribution and relationships of gender and age groups with the detection of SARS-CoV-2 RNA in the Two hundred and ninety-nine swab and saliva samples using the GeneFinder COVID-19 Plus RealAmp RT-PCR kit analyzed with the Biorad CFX96 Real-Time system.

Both nasopharyngeal swabs and saliva were positive in 20 (51.3%) participants, 10 (25.6%) had swab positive/saliva negative results and 9 (23.1%) had saliva positive/swab negative results (Table 3). The percentage of positive and negative agreement of the saliva samples as compared with the nasopharyngeal swab were 67% and 97% respectively. The positive and negative predictive values were 69% and 96% respectively.

**Table 3:** SARS-CoV-2 positivity by gender and sample type.

		Positive					
	Negative (%)	Positive for Both Methods (%)	Saliva Positive (%)	Nasopharyngeal Swab Positive (%)	Total (%)	X^2^	P value
**Gender**						**0.69116**	**0.8753**
Female	122(87.1)	8(5.7)	5(3.6)	5(3.6)	140(100)		
Male	138 (86.8)	12(7.5)	4(2.5)	5(3.1)	159(100)		
**Total**	**260**	**20**	**9**	**10**			
**Age Groups**							
**<20**	17(77.3)	3(13.6)	1(4.5)	1(4.5)	22(100)	**16.202**	**0.5785**
**20–30**	89(93.6)	3(3.2)	0(0)	3(3.2)	95(100)		
**31–40**	75(85.9)	4(4.7)	4(4.7)	4(4.7)	85 (100)		
**41–50**	48(85.7)	4(7.1)	3(5.4)	1(1.8)	56 (100)		
**51–60**	19(79.2)	3(12.5)	1(4.2)	1(4.2)	24 (100)		
**61–70**	9(81.8)	2(18.2)	0(0)	0(0)	11 (100)		
**>70**	5(83.3)	1(16.7)	0(0)	0(0)	6(100)		
**Total Positive**		**20**	**9**	**10**			

The relationships between SARS-CoV-2 positivity across the sample types (both nasopharyngeal swab and saliva samples, only saliva or nasopharyngeal samples), gender and age groups using the GeneFinder COVID-19 Plus RealAmp RT-PCR kit analyzed with the Biorad CFX96 Real-Time system.

**Fig 1:**
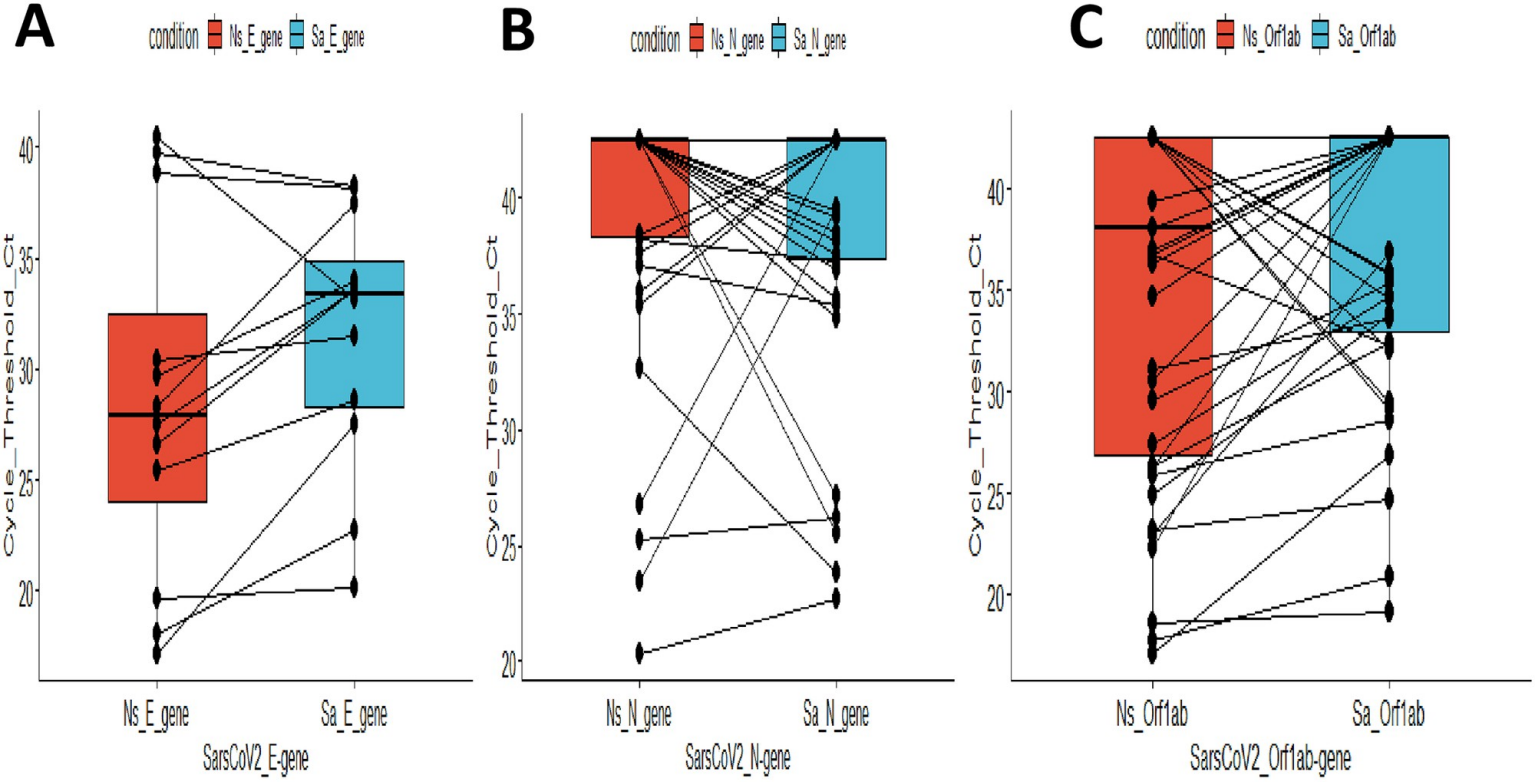
Cycle Threshold (Ct) values for SARS-CoV-2 positivity to (A) E-gene, (B) N-gene, and (C) Orf1ab gene respectively, show good comparison on both the Saliva and the Nasopharyngeal swab: The median Cycle Threshold (Ct) values for the three SARS-CoV-2 genes (E, N, and Orf1ab) of the positive samples (Ct <40) and the negative samples (Ct>40) from both the nasopharyngeal swab and saliva samples were compared using the Wilcon signed rank exact test. The p values of 0.07715, 0.8438, and 0.2293 were determined for the E, N, and Orf1 ab genes respectively. There were no statistically significant differences between the median SARS-CoV-2 E-gene, N-gene, and Orf1ab gene Ct values detected for the Nasopharyngeal swab and Saliva samples.

There was no statistically significant relationship observed between the sex/gender of the participants and their COVID-19 RT-PCR nasopharyngeal (p-value = 0.6042) or saliva test results (p-value = 1) using the Pearson’s Chi-Squared test for categorical variables. A strong agreement was observed between the Nasopharyngeal and Saliva methods which were statistically significant [Mean Square Contingency Coefficient (phi coefficient) = 0.64; p-value <0.01]. However, there was no statistically significant relationship between the age of the participants and their COVID-19 RT-PCR test positivity by saliva (p-value = 0.1512) and nasopharyngeal swab (p-value = 0.1066) respectively using the t. test for continuous predictor variables. Comparing the median Cycle Threshold (Ct) values of the three (E, N, and Orf1ab) genes of the positive samples from both the nasopharyngeal swab and saliva samples, shows there were no statistically significant differences between the median E-gene, N-gene, and Orf1ab Ct values detected for SARS-CoV-2 for both sampling methods (Nasopharyngeal swab and Saliva samples) (Fig 1).

## Discussion

The percentage of positive and negative agreement of the saliva samples as compared with the nasopharyngeal swab were 67% and 97% respectively. The positive and negative predictive values were 69% and 96% respectively. The findings from this study indicate that drooling saliva samples have good and comparable diagnostic accuracy to the nasopharyngeal swabs with a moderate percentage of positive agreement and a high percentage of negative agreement. Also, the saliva sample shows good Positive Predictive Values (PPVs), and Negative Predictive Values (NPVs). This suggests that the saliva sample has the potential as an alternate specimen of choice for a nasopharyngeal specimen for the expansion of access to COVID-19 testing in the country.

Presently in Nigeria, 5,441,162 samples had been tested according to the Nigerian Centre for Disease Control (NCDC) COVID-19 microsite [39]. This represents only about 2% of the over 200 million human population documented in the country. Although, since the first case of COVID-19 in Nigeria, various molecular laboratories had been established by the NCDC and stakeholders to expand access to COVID-19 testing across states, local governments, and rural communities. Resources for sample collection are very limited within the country. Thus, testing using saliva samples might be particularly useful in Nigeria which has an enormous population index, limited in-country testing capacity, dwindling economic resources, and political and immigration challenges.

Nasopharyngeal swab samples were generally regarded in many settings as the gold standard for SARS-CoV-2 testing by RT-PCR. The technical difficulties and gaps in know-how, procedural discomfort, risk of exposure (particularly to healthcare workers) and challenges with deployment to rural health facilities and communities are part of the numerous limitations of the use of NP swabs in communities and outpatient care settings [40–42]. The possible introduction of saliva samples into the Nigerian national algorithm for SARS-CoV-2 testing is further justified since saliva-based SARS-CoV-2 testing is already in use in some settings and the United States Food and Drug Administration has issued an emergency use authorization for a saliva-based protocol for SARS-CoV-2 testing [11–15].

The method provides a safe, viable, and cost-effective, sample collection method. Saliva seems to be a highly potential substitute for pharyngeal swab collection as it is easily gotten from patients who are expected to simply spit into a sterile universal container. As immigration restriction measures are relaxed with increased inter and intra countries interactions engineered by trade, commerce, and travel. Increased levels of sampling within our communities might become necessary for the continued surveillance of SARS-CoV-2 and the identification of new clusters of infections. The required increase in testing rates within our communities might only be achieved when alternative, simple, and readily acceptable sampling methods are introduced. Saliva sample collection provides an alternative convenient substitute with a comparable diagnostic efficacy even without the use of a transport medium as documented in this study. Hence, an understanding of the usefulness of saliva in a community-based screening setting is crucial, particularly in asymptomatic individuals.

Our findings aligned with multiple published works supporting saliva as an alternative sample for screening and diagnosis of COVID-19 [5, 9, 21, 42–49]. However, it was at variance with a few other reports where saliva was shown to be more sensitive than the corresponding NPS [50–52]. Participants recruited in this study were asymptomatic individuals residing within the low, medium, and high-density areas in Lagos, Nigeria. Several reasons may account for the discordance in the findings from various studies, which were not evaluated in this study.

Most published works using saliva samples for the detection of SARS-CoV-2 by RT-PCR have been carried out with the recruitment of patients with obvious COVID-19 signs and symptoms or other individuals with suspicious symptoms. While only a few studies had been carried out with cohorts of asymptomatic patients as done in our study. In groups of asymptomatic individuals, the lower sensitivity of saliva samples as compared to nasopharyngeal samples for the detection of SARS-CoV-2 RNA has been reported [42, 53–58]. Others with a higher sensitivity to saliva samples have also been documented [46, 54–62].

However, based on the context that the diagnosis of symptomatic and asymptomatic COVID-19 illness has several discrepant determinants depending on the type of clinical specimen type and variation in temporal viral shedding apart from the diagnostic primer/probe mismatches with infecting SARS-CoV2 virus sequence [9, 32, 45–48, 53]. The type of samples (saliva or nasopharyngeal swab) and the timing of collection for testing deserve careful consideration because these may affect the diagnostic accuracy of saliva and nasopharyngeal swab testing differentially [48, 50, 52, 54, 58, 61, 62]. Several studies that compared viral load in saliva and nasopharyngeal specimens have reported the detection of a higher viral load of SARS-CoV-2 in saliva than in nasopharyngeal swabs and for longer periods probably since ACE2 cells that cover the salivary gland ducts are the first target of SARS-CoV-2 [9, 32, 45–48, 52, 53, 58, 61, 62]. It is worthy to note that whether certain clinical syndromes influence the specific specimen types for optimal RT-PCR diagnostic accuracy, requires further investigations.

Our findings indicate that drooling saliva samples have good and comparable diagnostic accuracy to the nasopharyngeal swabs with moderate sensitivities and high specificities. However, the population studied were mainly young and middle-aged individuals who were either asymptomatic or had mild signs and symptoms but with the ease of use and good diagnostic performance, testing for the detection of SARS-CoV-2 using a saliva sample is recommended.

## Conclusion

Our study adds to the growing body of evidence supporting saliva as an attractive, alternative, sensitive, and less invasive sample collection method for SARS-CoV-2 RNA detection. It also highlights the possibilities of a self-collected saliva sampling method. With the added advantages of this alternate sampling approach being less invasive and technically less demanding, it would facilitate the efficient scaling up of SARS-CoV-2 testing capacities in community settings by enhancing acceptability and accessibility in a local community setting.

## Supporting information

S1 File Raw data and analysis. (XLSX)

S2 File (DOCX)

S3 File Saliva all positive figures. (XLS)
