# Supplementary material for: Saliva sample for detection of SARS-CoV-2: A possible alternative for mass testing
Source: PLoS One. 2022 Sep 28;17(9):e0275201. doi: 10.1371/journal.pone.0275201 (PMC9518879; doi:10.1371/journal.pone.0275201)
Supplement: S2 File — (DOCX) [file pone.0275201.s002.docx]

**SUPPORTING INFORMATION**

**Corresponding Author/Email: Olumuyiwa Babalola Salu** (Senior Lecturer/Medical Virologist). Centre for Human and Zoonotic Virology, Central Research Laboratory and the Department of Medical Microbiology and Parasitology, College of Medicine, University of Lagos, PM.B. 12003, Idi-Araba, Surulere, Lagos, Nigeria (Email: [obsalu@yahoo.com](about:blank), Mobile: +234 802 305 5077).

**Full Author’s Details:**

1. Olumuyiwa Babalola **Salu** (**Salu, OB**), (Senior Lecturer/Medical Virologist).

Affiliation(s) & Contact: Centre for Human and Zoonotic Virology, Central Research Laboratory and the Department of Medical Microbiology and Parasitology, College of Medicine, University of Lagos, PM.B. 12003, Idi-Araba, Surulere, Lagos, Nigeria ([obsalu@yahoo.com](about:blank), +234 802 305 5077).

1. Iorhen Ephraim **Akase** (**Akase, IE**), (Consultant).

Affiliation(s) & Contact: Consultant and Head of Infectious Disease Unit, Lagos University Teaching Hospital, Idi-Araba, Lagos. PM.B. 12003, Idi-Araba, Surulere, Lagos, Nigeria (akaseephraim[@yahoo.com](about:blank), +234 803 518 5940).

1. Mr. Roosevelt Amaobichukwu **Anyanwu** (**Anyanwu, RA**), (Laboratory Scientist).

Affiliation(s) & Contact: Central Research Laboratory, College of Medicine, University of Lagos, PM.B. 12003, Idi-Araba, Surulere, Lagos Nigeria ([palinalyze@hotmail.com](mailto:palinalyze@hotmail.com), +234 703 864 6302).

1. Mrs. Mercy Remilekun **Orenolu** (**Orenolu, MR**), (Laboratory Scientist).

Affiliation(s) & Contact: Central Research Laboratory, College of Medicine, University of Lagos, PM.B. 12003, Idi-Araba, Surulere, Lagos Nigeria ([mercy_seat03@yahoo.com](mailto:mercy_seat03@yahoo.com), +234 802 906 3035).

1. Mrs. Maryam Abiodun **Abdullah** (**Abdullah, MA**), (Laboratory Scientist).

Affiliation(s) & Contact: Central Research Laboratory, College of Medicine, University of Lagos, PM.B. 12003, Idi-Araba, Surulere, Lagos Nigeria ([aamaryam80@gmail.com](mailto:aamaryam80@gmail.com), +234 803 870 6081).

1. Temie **Giwa-Tubosun** (**Giwa-Tubosun, T**) (Chief Executive Officer)

Affliations: Research and Development Unit, LifeBank, 1, Connal Road, Yaba, Lagos, Nigeria ([temie@lifebank.ng](mailto:temie@lifebank.ng), +234 706 717 8618).

1. Sodiq Abiodun **Oloko** (**Oloko, SA**) (Director of Operations)

Affliations: Research and Development Unit, LifeBank, 1, Connal Road, Yaba, Lagos, Nigeria ([sodiq@lifebank.ng](mailto:sodiq@lifebank.ng), +234 703 791 4706).

1. Ayomide Michael **Oshinjo** (**Oshinjo, AM**) (Research Officer)

Affliations: Research and Development Unit, LifeBank, 1, Connal Road, Yaba, Lagos, Nigeria ([ayomide@lifebank.ng](mailto:ayomide@lifebank.ng), +234 813 183 4479).

1. Aisha Ajoke **Abiola** (**Abiola, AA**) (Chief of Staff)

Affliations: Research and Development Unit, LifeBank, 1, Connal Road, Yaba, Lagos, Nigeria ([temie@lifebank.ng](mailto:temie@lifebank.ng), +234 813 777 2557).

1. Kolawole Solomon **Oyedeji** (**Oyedeji, KS**) (Professor of Medical Laboratory Science).

Affiliation(s) & Contact: Department of Medical Laboratory Science, College of Medicine, University of Lagos, PM.B. 12003, Idi-Araba, Surulere, Lagos Nigeria ([kolaremi903@gmail.com](mailto:kolaremi903@gmail.com), +234 806 611 7865).

1. Sunday Aremu **Omilabu** (**Omilabu, SA**), (Professor of Virology & Director, Central Research Laboratory, College of Medicine, University of Lagos).

Affiliation(s) & Contact: Centre for Human and Zoonotic Virology, Central Research Laboratory and the Department of Medical Microbiology and Parasitology, College of Medicine, University of Lagos, PM.B. 12003, Idi-Araba, Surulere, Lagos Nigeria. Adjunct Professor, Centre for Human Virology and Genomics Research, Microbiology Department, Nigerian Institute of Medical Research, 6 Edmond Crescent, P.M.B 2013, Yaba 101012, Lagos, Nigeria ([omilabusa@yahoo.com](about:blank), +234 802 311 9431).
